# Cord blood banking – bio-objects on the borderlands between community and immunity

**DOI:** 10.1186/s40504-015-0029-8

**Published:** 2015-10-08

**Authors:** Nik Brown, Rosalind Williams

**Affiliations:** Department of Sociology, Science and Technology Studies Unit (SATSU), University of York, York, YO10 5DD UK

**Keywords:** Bio-objects, Biopolitics, Cord blood, Community, Immunity

## Abstract

Umbilical cord blood (UCB) has become the focus of intense efforts to collect, screen and bank haematopoietic stem cells (HSCs) in hundreds of repositories around the world. UCB banking has developed through a broad spectrum of overlapping banking practices, sectors and institutional forms. Superficially at least, these sectors have been widely distinguished in bioethical and policy literature between notions of the ‘public’ and the ‘private’, the commons and the market respectively. Our purpose in this paper is to reflect more critically on these distinctions and to articulate the complex practical and hybrid nature of cord blood as a ‘bio-object’ that straddles binary conceptions of the blood economies. The paper draws upon Roberto Esposito’s reflections on biopolitics and his attempt to transcend the dualistic polarisations of immunity and community, or the private and the public. We suggest that his thoughts on immunitary hospitality resonate with many of the actual features and realpolitik of a necessarily internationalised and globally distributed UCB ‘immunitary regime’.

## Introduction

Over the course of several decades, umbilical cord blood (UCB) has become the focus of intense efforts to collect, screen and bank haematopoietic stem cells (HSCs) in hundreds of repositories around the world. Units of UCB have been used effectively to treat a wide range of haematological and immunitary disorders particularly in reconstituting the blood and immune system following treatment for bone marrow malignancies and other cancers (Gyurkocza et al. 2010; Cutler and Ballen 2012). Cord blood has also attracted significant interest from the nascent research and clinical domains of regenerative medicine and efforts to harness the potential functional plasticity of stem cells. Our purpose here is to examine and follow umbilical cord blood units as ‘bio-objects’ (Vermeulen et al. 2012) that are constitutive of particular kinds of biopolitics, new magnitudes of scale, new dimensions of internationalisation that transform and reconfigure the conventional spatial and symbolic borders of the blood economies (Copeman 2009).

The UCB banking sector has developed through a broad spectrum of sometimes distinct and sometimes overlapping banking practices, sectors and institutional forms. Superficially at least, these sectors have been widely distinguished in bioethical and policy literature between discursive notions of the ‘public’ and the ‘private’, the commons and the market respectively (Waldby 2006; Brown and Kraft 2006). The former is supposed to gesture towards a sector in which cord blood is potentially available for relatively free circulation within the blood economies of the world. The latter is often used as a pejorative shorthand for forms of banking in which UCB is retained privately in ‘family banks’ by paying clients. Although, as we go on to show in our discussion below, these differentiated discursive frameworks are far from straightforward in practical, material and institutional terms.

The rhetorical and moral worlds with which both of these domains are associated have been marked by an acutely sharp set of binary bioethical distinctions. ‘Public’ banking is frequently seen to operate on the basis of a freely donated solidaristic gift economy in which commonly held assets are available for treatment without privilege or preference. ‘Private’ banking on the other hand is often seen to reference a retreat into the sphere of personal property, a form of enclosure in which assets are withdrawn or diverted from circulation and availability (Fannin 2013). Most policy and bioethical discourse has been shaped by this highly dualistic framing of the public and the private, contrasting the commons with isolates, and can be illustrated by this frequently cited bioethical intervention from 2004:

*Tissue banks were up till now relying on free donation for treatment to the benefit of other persons or for research, and by the fact that it implies an act of solidarity or generosity it contributes to the social cohesion, while the commercial cord blood banks are running for profit. This reflects a more general shift to a privately funded health care system from a health system based on solidarity and motivated by public health considerations, which has characterised Europe in the last decades* (European Group on Ethics in Science and New Technologies, 2004, 1.22)

There has already been some attention given to the complexity and sociological significance of ‘private’ or ‘family’ cord blood banking seeking to go beyond the pejoratives of individualism, self-interest and the profit motive (Brown et al. 2011). But our purpose in this paper is instead to reflect more critically on the sentiments and discourse of ‘public’ cord blood banking. In so doing, we want to explore and examine key fundamental divergences between a binary bioethical and policy discourse compared and contrasted against the practical realpolitik of the ‘public’ cord blood banking and clinical world.

Our discussion is developed by reflecting on the dominant overriding bioethical discourses with which the public banking sector is associated. These discourses have traditionally configured the blood economies through notions of gift and altruism, the community and the commons, national solidarism and belonging, insularity from the market, and a discourse that presents donations as surplus ‘waste’. By contrast, our paper follows cord blood as a remarkably uneven ‘bio-object’, an object that straddles and hybridises the borderlands through which it travels. ‘Bio-objects’ have been loosely defined as phenomena that unsettle and subvert social, epistemological and regulatory boundaries in exactly the way discussed here (Vermeulen et al. 2012). Indeed, bio-objects like UCB can be ‘… characterised as having considerable fluidity and mobility across different socio-technical domains’ (Webster 2012: 3). Such objects can be seen to increasingly confound, migrate and reconfigure the political, spatial and economic dimensions of the life sciences. Indeed, cord blood in particular might suitably be described as a ‘boundary crawler’ (Holmberg and Ideland 2012) unsettling many of the customs and beliefs attributed to blood, donation and giving.

Theoretically and conceptually we also want to situate our critique in a growing body of biopolitical literature on the ambivalent meanings of, and relationships between community and immunity, or *communitas* and *immunitas* (Esposito 2008, 2010, 2011; Sloterdijk 2011; Cohen 2009). Our key intellectual reference point in this discussion is Roberto Esposito and his thoughts on what he calls an ‘immunitary paradigm’ or ‘regime’ together with his critical perspectives on the origins and social fabrics of community, gift and solidarism. In this respect we also want to revisit but also considerably expand upon a characterisation of cord blood HSC banking as a form of ‘immunitary bioeconomy’ (Brown et al. 2011) in which the worldwide banking, global distribution and circulation of UCB HSCs are traced through a spectrum of logics that are neither necessarily ‘public’ nor ‘private’ per se.

We explore the construction of an immunitary regime premised on an ‘allogeneic logic’ where units of umbilical cord blood are made available through international circuits for transplantation between closely matched donors and recipients (self-to-other). In so doing, we show how UCB banking and treatment transcends traditionally received values of the blood economies bisected between gifts and markets, between community solidarism tied to nationhood and atomistic self-interest tied to the market. Instead, blood and especially umbilical cord blood, can be seen to subvert and destabilise both ‘imagined nationhood’ on the one hand, and ‘imagined markets’ on the other. Cord blood banking, at least in its ‘public’ form can, we suggest, be seen to fracture and fragment the totalising or hermetically insulated biopolitical domains of community and immunity, the public and the private, the gift and the market, the other and the self.

In this respect we examine how this articulates with Esposito’s conception of an affirmative immunitary biopolitics which similarly offers the potential to transcend the binary polarisations of pure community and pure immunity. We suggest that his thoughts on immunitary hospitality resonate with many of the actual features and realpolitik of the necessarily internationalised and globally distributed UCB immunitary regime.

Empirically, our approach in this discussion is informed by sustained social scientific engagement with the worlds of umbilical cord blood banking internationally and spanning a decade or more. This comprises scores of interviews, focus groups, market assessments, UCB bank site visits, grey literature reviews and documentary analysis, together with other forms of empirical enquiry. Our respondents, all anonymised, span a broad range of stakeholders including donors and depositors, clinical professionals, banking personnel, policy makers and commercial actors.^1^

In what follows we first begin with a brief overview of Esposito’s inquiries into the biopolitics of *communitas* and *immunitas*. We then take these reflections forward to explore a number of dimensions of UCB banking, each of which can be seen to disturb some aspects of the dominant conventional discourses of ‘public banking’ as a straightforward expression of solidarism insulated from the market, and as waste to be freely donated.

### Immunitary life – between *communitas* and *immunitas*

For Esposito, community and immunity have their common etymology in the *munus*, roughly translating to mean gift, or obligation, and expressing the mutual obligations of the dutiful bond, of gift giving and reciprocity. ‘Nothing seems more appropriate today’ he writes, ‘… than thinking about community; nothing more necessary, demanded and heralded by a situation that joins in a unique epochal knot the failure of all communisms with the misery of new individualisms’ (2010: 1).

And yet his account contrasts starkly with most if not all sociological understandings where community has too easily been conceptualized through a romantic or nostalgic imagining of the commons. In sociological thought, community becomes that which provides or furnishes belonging, a sometimes territorially shared sense of identity and identification to which individuals form subjective affective attachments. Community is, in these accounts, a good, a property, a value. That which is common unites. This formulation extends through, but is not confined to, Tönnies (1957) and other sociological formulations of community. Esposito contrasts his own thinking to that of these more intuitively familiar and well-known understandings. How is it, he asks, that we naively came to conceive of community as that which enriches whilst immunity deprives? ‘What’ he writes, ‘is the ‘thing’ that the members of the community have in common, and is it really ‘something’ positive? Is it a good; is it a wealth?’ (2010: 6). In tracing community to the *munus*, Esposito offers a radically different formulation where the *munus* implies the constant potential of a ‘diminishment of one’s own goods and in the ultimate analysis also of oneself’ (Esposito 2006: 50).

The source of community, emerging from the *munus,* is an unsettled obligatory indebtedness, a deficiency and lack, a void-like absence that must be filled, compensated for or repaid. The fact that community in its original form is driven by this potentially insatiable deficit crucially distinguishes Esposito’s thinking from sociological theory: ‘… the *munus* that *communitas* shares isn’t a property or a possession. It isn’t having, but on the contrary, is a debt, a pledge, a gift that is to be given, and that therefore will establish a lack’ (2010: 6). The crucial question here becomes one of understanding the different forms taken in different periods by the means deployed to set limits on the insatiability of community.

For the solution to this problematic, Esposito turns to the related term *immunitas*, a freedom or protection from the *munus*. Immunization is of such profound significance, he writes, ‘that it can be taken as the explicative key of the entire modern paradigm… even more than… ‘secularisation’, ‘legitimation’ and ‘rationalisation” (2010: 12). *Immunitas* develops to counter an all-encompassing reciprocity, to provide protection from obligation and insulation from the potentially unappeasable commons.

Immunity is, for Esposito, the underlying logic of the modern political order, particularly in the contexts of property, individualism and securitization. Life and politics are intimately interwoven in an immune system rationality that is both corporeal and institutional. In both medical and juridical discourse, immunity is a form of exemption, protection or untouchability. At its simplest and most straightforward, *immunitas* is defined as that which ‘protects the one who bears it from risky contact with those who lack it’ (2008: 50). Immunity therefore safeguards life whilst at the same time setting strict limits on life, curtailing its exposure to contagion, depletion, risk. ‘If *communitas* is that relation, which in binding its members to an obligation of reciprocal donation, jeopardises individual identity, *immunitas* is the condition of dispensation from such an obligation and therefore the defense against the expropriating features of *communitas’* (2008: 50).

The biopolitics of immunization does not necessarily refer to the conditions or the external circumstances of the body but instead extends into and from the body. In this way, the immunities of politics, on the one hand, and those of the body on the other, overlap and interpenetrate. In the context of literature on bio-objects, Metzler makes just this point arguing that politics do not ‘come after the bio-object’ but instead must be read as ‘assemblages that may stabilize vital phenomena as bio-objects’ (Metzler 2012: 152). Esposito echoes this point in writing that the only ‘politics possible’ is one ‘inscribed in our natural code… politics remains in the grip of biology’ (2008: 24). For Esposito however, life is not invested with an immunity laid over life. Instead, immunity *is* life: ‘Rather than arguing that power becomes ‘joined’ to life, the term ‘immunity’ will enable us to describe the concurrently enhancing and proliferating, and toxic and auto-negating properties of the ‘power to preserve life” (2008: 46). As we go on to show with reference to UCB banking, the central bio-object of the immunitary paradigm is the very immune-system vitality of the body itself.

Esposito is keen to point to ways in which immunity creates the conditions for and facilitates new forms of circulation, movement and exchange. In his writing, immunity and community are far from polarised with complex interpenetrations in which some forms of immunity can lead to productive forms of association, flow and ‘immunological tolerance’. Writing of immunity’s place in modern biology, he asks, ‘isn’t it precisely the immunitary system… that carries with it the possibility of organ transplants’ (Esposito 2006: 54). Biomedicine is linked therefore to a ‘non-negative, hospitable’ immunity (*ibid*: 54) within an affirmative biopolitics becoming ‘the power to preserve life’ (2008: 46).

Immunity is explicitly far from rigid or impermeable within his formulation to the point of necessitating transgressive breaches. He cites approvingly Durkheim’s reflections of vaccination as illustrating the way immunity requires a tolerance for more fluid contact across the barriers and boundaries of protection: ‘… smallpox is a true disease that we give ourselves voluntarily, yet it increases our chance of survival. There may be many other cases where the damage caused by the sickness is insignificant compared with the immunities that it confers upon us’ (Durkheim cited in Esposito 2008: 48). The immunitary protection of life therefore, depends paradoxically upon a requirement to relinquish or sacrifice a pure and hermetically sealed self so that ‘… in order to be saved, life has to give up something that is integral to itself’ (2008: 59).

These reflections offer a very different and contrasting formulation of the relationship between *communitas* and *immunitas*, one where the binaries (between the market and the commons, the individual and the collective, etc.) are less straightforward. To this extent, Esposito chimes with and echoes very similar observations and tensions in more recent writing on the bioeconomy and the commercialisation of the body, pointing to the inadequacy of simplistically naïve dualisms between commodity and gift, use and exchange value (Waldby and Mitchell 2006; Hoeyer 2009; Brown 2013). *Communitas* and *immunitas* can therefore be seen to contrast markedly against, rather than align directly with, the traditional binaries of the blood economies expressed by Titmuss’ ‘gift relationship’ (1970).

### Between the cosmopolitan and the national

According to the World Marrow Donor Association (WMDA), over 40 % of UCB units released for treatment are either imported or exported across international borders (Welte et al. 2010). More usually, units are internationally couriered by flight transfer in mobile cryogenic flasks. In a recent field trip to a cord blood stem cell bank in Madrid, we were able to follow the process of releasing a unit for treatment. Close to the exit of the bank there is a small area scattered with what look like old milk urns. These are knee-high aluminium liquid nitrogen containers used to transport UCB units to treatment centres around the world. Each one is battered and, like old suitcases, they are littered with travel documents and labels and other signs of travel. There are envelopes, barcodes, document bags, security permits, flight tags for Swiss Air, United Emirates, Pan Am, and other airlines. One of the labels bears the address of a cancer treatment centre in New Zealand along with large yellow stickers reading ‘DO NOT X-RAY’. Once in travel, UCB might be said to enjoy a certain kind of diplomatic immunity or exemption from the strictures of international bio-security.

At any one time, one or more of the bank’s units will be travelling by international courier to a destination hospital somewhere else in the world. In the previous week, a match had been found for a patient in New Zealand. That empty container had now been returned and lay at our feet here in Madrid’s cord blood stem cell bank. Cord blood banking is, in these terms, coextensive with immunitary migration, heterogenisation and dispersion. By extension, banks such as this sit awkwardly with blood’s communitarian connotations of civic indebtedness tied so closely to the nation state (Copeman 2009).

The UCB immunitary regime depends upon access to, and penetration of, hundreds of thousands of stem cell units accumulating internationally in facilities just like this one in Madrid. All are inter-linked and synchronised through real-time registries, vast computational databases, meticulously recording the specific immunological (HLA) typology of each individual unit. Lupton (2015) similarly writes of the significance of code, informaticisation and metrification in the assemblage of modern bio-objects, a key characteristic of which is the production of ‘data doubles’ creating new patterns of correspondence between data points and bodies. Such is the scale of this doubling that there are now in excess of half a million (601,316) units of UCB coded and registered as available for treatment globally (World Marrow Donor Association 2013: 19).

Cosmopolitan internationalisation is therefore central to the underlying rationale and purpose behind the establishment of the UCB immunitary bioeconomy. Cord blood banks are different to bone marrow registries and make available a very different kind of immunitary population of HSCs. Registries list willing donors and predominantly draw from a pool of largely ‘white’ or ‘Caucasoid’ donors, the traditional demographic mainstay of the Western blood economies. Beyond the largely ‘white’ donor pool of the Western blood economies, the chances of finding a match for a non-white leukaemia patient diminish considerably. This can decline from a 70 % chance of finding a match for Caucasoid transplant recipients to as low as 20 % or less for non-Caucasoid patients (Meijer et al. 2009). Bone marrow registries tend to reflect or mirror forms of mainstream demographic identification and belonging linked to sentiments of ‘national’ rather than ‘minority’ cultural identification.

Much of the ‘public’ UCB banking sector has therefore been established to replenish and populate what amounts to an immunitary vacuum within the racial and HLA composition of the existing bone marrow registries. Without the gigantic scale and interpenetrative reach of registries and banks, the chances of finding a match would be vanishingly small (see Williams 2015). This global cosmopolitan scale reflects the requirement for immunitary specificity, just as it also reflects immunitary rarity and the infrequency of immunities.

Cord blood banking registers a particular set of immunitary relations where the individual immune system interpenetrates with the establishment of a globalised infrastructure for mobilising, circulating, trading and storing immunitary assets in the form of HSC units. Drawing on another key immunitary theorist, Peter Sloterdijk (2011), we might say that the ‘micro-spherologies’ of individual immunity can be seen to coalesce and potentially intermingle with the ‘macro-spherologies’ of a supranational cosmopolitan biopolitical infrastructure.

Banks are required to segment, isolate, discriminate and characterise at a level of molecular immunitary detail possibly far more exacting than in other areas of transplantation. Finding exactly the right match between one immunity and another requires the creation of these super-massive economies of scale. This molecular specificity is then projected onto and into globalised immunitary connectivity operating through molar levels of association.

With an unintended, though apposite, play on the theme of blood, Esposito argues that *immunitas* is capable of becoming ‘… the coagulating point, both real and symbolic, of the entire contemporary experience’ (Esposito 2006: 51). That ‘coagulating point’ precisely expresses a regime that requires and makes possible molecular specificity, but through its globally molar scope and span. Here then, regional attachments and sentiments of communitarian mutualised indebtedness are perforated by forms of international immunitary exchange and intermediation.

The realities of the globalised UCB immunitary infrastructure therefore operate in stark contrast to received and romantic notions of blood as the basis for the Titmuss-like values of civic belonging and national identification. The development of the blood economies during the course of the twentieth century have been directly tied to these notions of ‘citizenship, solidarity and imagined national communities’ (Busby et al. 2013: 83). Whilst blood has become an established medium of a commonality put to work in the defence of the nation, this can be seen to collide with a far more internationalised ‘haemato-global assemblage’ (Ong and Collier 2005; Simpson 2014).

The UCB bioeconomy troubles and destabilises traditional and coherent notions of community in a number of profound ways. Most UCB banks have been established on the basis of a logic of radical diversification. That is, the more varied and heterogeneous the banked units, the more valuable and clinically significant is the collection. As we note above, established bone marrow registries, for example in Australia (Samuel et al. 2007) the United States (Johansen et al. 2008) and the United Kingdom (Brown et al. 2000), are over-represented with white donors, the customary blood donor population of many Western nations. In this sense, the bone marrow registry mirrors a certain kind of ‘imagined community’ (Anderson 1983), one that has been so essential to cultures of blood donation with its cultural and symbolic roots in notions of nationhood and patriotism (Waldby and Mitchell 2006). But some bloods (particularly cord blood) transverse the traditional borders of community in its nation-state formulation. This is an immunitary regime which must necessarily be open to international connectivity, where the travel of regenerative tissue and economic exchange can be plotted across countries and continents in ways Titmuss (1970) would never have imagined.

But the cord blood immunitary regime is also coextensive with very particular, locally articulated, geographical distributions of colonial history, migration and movement. Cord blood represents ‘not a rupture with colonial dispositions’ (Anderson 2014: 382) but one which is predicated on an immunitary reconfiguration of colonialism. It remains common in the context of the clinical and scientific discourse of UCB HSC banking to formulate cord blood through the language of ‘ethnicity’ and ‘race’ with categories like ‘white’ and ‘black’, the ‘Caucasoid’, the ‘Caucasian’, the ‘oriental’, ‘Chinese’, ‘African’ and ‘African-American’. HSC scientific discourse moves freely between arcane racial terms like ‘admixture’ and ‘heritage’ to ‘breeding’ and ‘outbreeding’ (Brown et al. 2011) and conceptual slippage into racialised discourse (Bliss 2011). This then is a regime taking a molar internationalised form, but generated through highly localised nodes of often idiosyncratic collection practice.

Nonetheless, it has become crucial for regional banks, and the international registries that connect them, to reassemble this globally distributed diasporic immunity. In this sense, UCB banking indexes and reconnects the remote immunitary contours of migratory globalisation and the spatial and generational flow of widely dispersed immune system life. Banks and registries thread their way around the world with a necessary requirement to establish an interconnected web of cross-referenced immunities.

What starts to emerge when focussing on cord blood as a bio-object is this proportional relationship between the molar and the molecular. That is, just as the immunotyping of our bodies becomes more specific, with ever-greater levels of ‘resolution’, the broader and wider becomes the ‘pool’ of available immunitary resources. As such, it is less and less possible to conceive of tissue donation within the terms of national solidarism, the imagined community of the nation state.

The statistical probability of establishing a match between a donated unit and a recipient depends upon vast economies of scale. With the potential for incredible variation between each immunitary type, the chances of finding a match are vanishingly small and only improve with access to more units, and a greater immunitary diversification of those units stored. The minutiae of molecular immunity needs to interpenetrate in this way with the global molar in order for matching to work. The larger and more heterogeneous the collection, then the greater the likelihood of “matching” the otherwise globally disconnected immunities of the unit and host. Again, these magnitudes of scale are key features of contemporary bio-objects which require ‘… the construction of large-scale international scientific collaboration and the transformation of government’ (Vermeulen et al. 2012: 172).

Whilst the moniker of the ‘bank’ implies something static or motionless, banks like the one in Madrid signal new forms of immunitary dynamism and potential for the acceleration of circulation and flow. HSC transplantation has traditionally been serviced by bone marrow registries listing possible donors. Cord blood banks on the other hand accumulate HSC *donations* rather than *donors*. There are over of fifty thousand stem cell transplantations performed annually (Gratwohl et al. 2010). Although the majority are sourced from registered bone marrow donors, a growing proportion are increasingly sourced from previously collected umbilical cord blood (Cutler and Ballen 2012). In 2012, 4150 cord blood units were issued internationally for treatment (Celluzzi et al. 2014). This fact alone is important in registering the fundamental shift in an immunitary logic transitioning from a registry of possible immunities, to a banked collection of materially tangible immunities. One is an immunitary resource *in potentia* while the other is a resource *in actu*. In this sense then, ‘… cord blood is more amenable than bone marrow to off-the-shelf and on-demand availability and circulation within a time sensitive system of distribution and exchange’ (Brown et al. 2011: 1116).

Under the sometimes pressing temporal circumstances of clinical need, UCB banks promise ready mobility and accelerated circulation. While bone marrow extraction depends upon complex negotiations with donors to arrange further tissue typing, lengthy apheresis processes, or invasive surgical extractions, UCB collection is presented as far more straightforward. Though, as we go on to discuss below, collection itself is not without its own burdens for those who wish to donate or deposit. The point here is that UCB banking represents a different kind of immunitary regime to that of the bone marrow registry, signalling towards a system of accelerated global circulation premised on stockpiling immunities poised for ready mobilisation.

### Between gifts and markets

In addition to profound internationalisation, there are also other realities to the immunitary regime of international cord blood banking that contrast starkly with traditional notions of the commons, and particularly a community insulated from the circulations of the market. Gift and donation occupy an acutely ambivalent position within modern bioeconomies. ‘Public’ sector banking operates in highly variegated ways, and according to prevailing principles of new public management, healthcare marketization and ‘privatisation’ in its many varied forms (Waldby 2006; Cooper 2008; Hoeyer 2009).

The values articulated in altruistically gifted UCB donations are in tension with the fact that cord blood units almost always, in one way or another become bearers of monetary value, price and cost. The notion of ‘cost’ is heavily layered and rarely more so than in the context of a discussion about blood, organs or other human tissues. Units carry the costs of extraction, initial diagnostics, typing and storage. This is usually somewhere in the region of a few thousand euros. These costs can become the basis for establishing and projecting market worth. For example, assuming that each banked unit around the world ‘costs’ around two thousand euros, the amount ‘spent’ on storage globally is somewhere in excess of a billion euros (1.1bn) or more. And UCB units cost when folded into the overall expenditure attached to a clinical intervention. ‘Pricing’ can take the form of a particular audit or cost code through which treatment centres may be reimbursed for a service, like any other form of treatment. In the UK, where the National Health Service (NHS) absorbs individual treatment costs on behalf of patients, these are usually referred to as ‘health resource groups’ (HRGs). Of all HRGs, cord blood transplantation (‘currency codes’ SA22A and B) rank among the most expensive single NHS treatments available alongside the likes of heart and lung transplantations. In fact, HSC transplantations, from whatever source (adult donor or UCB), account for eleven of the twenty most expensive NHS HRG currency codes.

Units also cost when released for treatment by banks. The Madrid UCB bank discussed above levies a ‘flat rate’ of 21,000 euros for the release of a UCB unit irrespective of whether the intended recipient is based domestically or overseas. Most other countries charge a considerably higher rate if the unit is to be exported. This can be anywhere towards 40,000 euros or more. The ‘flat rate’ operated by Madrid is possibly unusually distinctive and reflects the structure of a mosaic nation-state where the regionalisation of its healthcare systems means that almost everywhere is somehow international.

One of the fundamental tendencies of markets is they create the circumstances within which it becomes possible to exploit differences in value, differences in cost. It is the high costs of importing cord blood from overseas that has proven to be a powerful incentive for regional and domestic health services to establish their own supplies (Williams 2015). This is not to arrive at a point of internal self-sufficiency where a bank might be expected to entirely supply its own domestic requirements for cord blood. Rather, it is to arrive at a point where ‘stocks’ and ‘supplies’ are sufficient to derive an economic benefit by exporting high value units to other countries. But just as crucially, if not more so, no single bank is ever likely to attain a sufficient scale with which to satisfactorily meet domestic demand. As we have shown above, cosmopolitan internationalisation underpins the very possibility of maximising the statistical probability of matching a recipient host immunity to an available unit. The only exceptions here might be some East Asian countries, notably Japan, where the internally homogeneous composition of some populations results in a more internally-oriented supply chain (Takanashi et al. 2011). But for the most part, retreat into the micro-spherology of the region or the nation is largely irreconcilable with the heterogeneous global diversification and distribution of immunitary relations:

*‘…the HLA is so polymorphic that no country would be able to think itself sufficient even with the largest bank … you need the international collaboration. we’re maximising the probabilities of finding a donor … we are all fully aware that we will be providing for… abroad as indeed benefiting from … other registries… the figures with export/ import are quite clear… this is an international collaboration…’ (Director of a public UCB bank 1).*

So, an income can be made on exporting a unit overseas. That income can counterbalance the similar costs of importing a unit. It can also diminish the expense of running a bank. But in most circumstances it is not strictly speaking a source of profit or surplus since it is rarely likely to exceed investment costs. In this way, banks can offset at least some of the costs of collection, processing and storage within a complex balance of trade between internationally distributed participants. Cord blood therefore *costs* and is the bearer of monetary worth and value. But the notion of ‘profit’ or the ‘profit motive’ plays very little part in this discourse. Instead, the discourse used in ‘public banking’ is that of ‘off-setting’ and ‘compensation’. Internationalisation is a fundamental prerequisite of this immunitary regime, which in turn generates the potential for a price (in the form of ‘costs’) to be folded into the arrangement to release a unit for treatment. It is intended that this is not to generate a ‘profit’ as such, but to mitigate the economic risks and financial burden of domestic collection and storage.

This is a picture that is disruptive of both *communitas* and *immunitas* and their respectively affirmative and negative overtones. Although the cord blood bioeconomy may appear to be a market, it is not. Nor is it the freely unrestricted circulation of altruistically donated gifts*.* It is in this sense that UCB banking subverts both the moral economies of the gift, and the political economies of the market. In other words, it subverts the solidaristic-romanticism of a particular version of community, and the market-despotism of a particular version of immunity. As Hoeyer expresses it, the body is an unusual site in which circulation and flow can often come to depend on negotiating a price, ‘without forming a ‘market” (2009: 239). ‘Compensation’ is a standard monetary discourse in the tissue economies for simultaneously moving both money capital and tissue capital in a way that guarantees flow, but without resulting in overt money-profit. Very few human tissues can be said to be ‘commodity’ things, in the sense that say grain, ore or oil are. Prices too are often fixed at a somewhat arbitrary rate that is considerably below actual investment costs. Money takes unusual market forms in the tissue economies, often verging on little more than a form of ‘recognition’ or ‘acknowledgement’ between trading parties (Hoeyer 2009).

That said, UCB banking operates according to an economy of qualities (Callon et al. 2002) in which it becomes possible for treatment centres to choose and discriminate between the contending units on offer around the world. Those involved in structuring banks do think of themselves in the terms of operational businesses with products of varying quality on offer in a quasi-marketplace of sorts. And like most businesses seeking to occupy a market niche, they can find themselves combining sometimes contradictory strategies:

*‘… we have been very successful… forty percent of our collection is from ethnic minorities. There has been a price that we’ve paid for that in terms of business because we’ve shown that those from ethnic minorities have lower volume and lower TNCs [Total Nucleated Cell count]. So a large number of our units are considered not the optimal product. That’s the price we’ve paid … so from the business point of view we’ve not been all that successful in selling them as it were…’ (Director of a public UCB bank 1).*

Many of these features, and those of the wider blood and tissue economies, sit awkwardly with binary notions of the market on the one hand, and standard accounts of community or the commons on the other. Neither of the twin bioethical totems of gifts and commodities come near to appropriately capturing the logics of the UCB immunitary bioeconomy. UCB is the basis for an internationalised form of circulation made possible by forms of transaction that involve distributions of quality, price, scarcity and availability.

And yet, these realities are in evident conflict with the fundamental features of a moral economy underpinned by the notion of ‘the gift’. The prohibition on attaching a price by way of paying, or being paid, for one’s blood is enshrined in law in various jurisdictions (in the EU see 2004/23/EC) and has increasingly become established as a mainstay of the blood economies (Copeman 2009). However, dutifully ‘given’ units of cord blood become sites of trade and value investment that look very different to the reified the attributes of the gift economy. On the other hand, those features of trade and exchange also look very different to pure commodity markets. Instead, the immunitary regime of umbilical cord blood banking operates in an indefinable hybrid zone that subverts both ‘the market’ and ‘the gift’.

So the prohibition on trade in human matters results in particular forms of exchange or transfer (compensation, off-setting, acknowledgement, etc.). But it can also create disturbances in the moral fabric of the voluntaristic gift economy upon which those exchanges are built. For donors, the possibility of a ‘market’ where there should not be one is and can be a source of acute ambivalence (Healy 2006).

It becomes important to ask whether, instead of generating flow and an openly distributed reciprocity, the principled ideal of *communitas*, looks like regional localisation, a limit, a constraint, a potential restriction, a looming deficiency? Notions of the gift and the public may be seen to operate in rigidly insulated and principled isolation, blinkered from the immunitary world of international movement, of trading, transfer, business models and strategies. In its present formulation in the blood economies, is it possibly the case that the gift has the form and features of, in Esposito’s terms, a ‘diminishment of… goods and … also of oneself’ (Esposito 2006: 50)? There is, then, scope for re-thinking and possibly restructuring a discursive polar conflict between, on the one hand, a superficially idealised *communitas*, and on the other hand, a superficially idealised *immunitas*.

### Between waste and value

We now want to move towards a more critical reflection on the *munus*, the gift itself and what it is that is given, sacrificed or relinquished in the process of cord blood banking. Like other fields of the bioeconomy, UCB banking across all its sectors draws upon a common articulation of the blood of the umbilical cord as surplus ‘waste’. The category of waste here implies the straightforward gift of something that is either self-replenishing (in the case of peripheral blood) or something that would otherwise be discarded (in the case of the umbilical cord). The umbilical cord and the placenta are discursively framed as a disposable by-product of the birthing process with invaluable future clinical potential in one of a wide range of life-saving applications (Brown 2013; Santoro 2009). This discourse of waste, as a classificatory register, imposes a powerful moral injunction not to squander something potentially precious.

Waste also serves to defuse potential conflicts over property and possession (Healy 2006). In other words, it is simpler to transfer ownership, if the transfer involves a movement from one for whom something has *no* value, to one for whom it *does* have value. It is then, a gift which incurs no sacrifice or expense, no *munus* as such. Taken to its logical conclusion, the absence of sacrifice may even invalidate this kind of giving as a true gift. The giver has nothing to lose by giving nor does the giving entail a cost or diminishment for the donor. There is in this case, as Esposito might put it, no lack that follows from the gift. Waste in these terms is an ethically loaded accusation, a rhetorical space for allegation. It specifies something as potentially lost, misused, unexploited, and left idle or vacant. It is a vacuum or void to be filled by a new obligatory purpose, a new use. Waste formally presupposes a use or purpose to which something could be put, if only it were released from the category of the wasted. This configuration of UCB as waste, as an otherwise unwanted and unrequired surplus or excess of birthing, is the first step in decoupling blood from its source (Brown 2013). Collection rests on these discursive notions of the non-invasive convenience of the procedure, its lack of cost, particularly in comparison to bone marrow extraction or peripheral blood apheresis.

However, while waste may be presented as something that should be a matter of passive indifference to the donor, it is far from it. The collection of cord blood is not without serious contention, taking place amidst the many competing clinical demands of pregnancy and the birthing process (Royal College of Obstetricians and Gynaecologists 2006). Potential donors or UCB depositors can become finely attuned to contesting the dominant discourse of waste in UCB banking. The umbilical cord is increasingly a site of acutely competing biopolitical tensions with fundamental implications for the UCB bioeconomy (Brown and Kraft 2006; Dickenson 2007; Waldby 2006; Brown 2013). This is especially the case with respect to, for example, the timing of cord blood clamping. The instant at which the umbilical cord is clamped and cut in the moments after birth has become highly contentious. In most modern medicalised birthing contexts, it has become common practice in recent decades to execute cord clamping immediately upon delivery of the infant. By coincidence, these are also the ideal conditions for extracting very high volumes of blood from the umbilical cord. The shorter that interval between birth and clamping, the higher the ‘yield’ of UCB stem cells.

But this intertwined relationship between collection and immediate clamping is now deeply contested by those who have argued that a delay in the clamping of the cord is necessary for neonatal health. The World Health Organisation has advised that there should be a delay of three minutes before clamping (World Health Organisation 2014) with similar guidance having been issued by other professional and health sector bodies (Royal College of Obstetricians and Gynaecologists 2006). Crucially and paradoxically, it is the very promotion of the UCB donation and banking that has itself revived and refocused a broader debate about cord blood clamping (Brown 2013). The discursive register of waste has, therefore, reinvigorated a competing register of value and the practice of delayed clamping. For potential donors, it is the value of umbilical cord blood to the banking sector that inspires them to think more critically about its value to their neonates. What follows is a short extract of focus group conversation convened with expectant parents:

*Sarah: The … thing that put me off [donation] was one article about cutting the cord very quickly … if they don’t cut it for 10 minutes it can be good for the baby…*

*Florien: This is something I didn’t consider … I just thought you cut it and then there’s something left inside … I think it’s a bit stupid to cut something off to save for later if it could be used now. So if what is left afterwards can be of some use then it’s fair enough.*

*Sarah: We kind of all thought of it as a bit of by-product … rather than it being still of some relevant value at that point.*

*Florien: …some of it is left really but they want more than just a small bit. They prefer a good bit.*

The void or vacuum at the heart of a discourse of waste is a powerful device implying the existence of a latent value at risk of going unrealised. In this and other conversations with expectant parents, the register of waste gives potential donors a window through which to envision value where it may not before have been. Waste essentially equips parents with an awareness of the tissue’s more immediate potential for vitalistic regenerativity in their own neonate. The future-latent purpose or value of CB banking undergoes a radical inversion or reversal, now refocusing away from the gift towards possession, away from future latency towards the immediate, from *potentia* towards *actu*. In Esposito’s terms, the possibility of a donation becomes a potential endangerment, a genuine sacrifice, a *munus*.

To revisit our point above, gift is not without its costs. Like many aspects of bioeconomy, gifts can come to depend on the potential for some kind of forfeiture and sometimes (self-)harm. As such, gifts can be sites of ambivalence, contestation and circumspection in which a discourse of waste suddenly looks weak and unconvincing. The idea that the giver has nothing to lose becomes a very thin veneer over underlying and contending registers of value.

Like most forms of blood donation, it temporarily or otherwise depletes life, as well as potentially saving life. Blood donation, for example, is accompanied by a long list of usually minor adverse effects including nausea, light-headedness, faintness (Sojka and Sojka 2008), tachycardia, perspiration, fainting (Masser et al. 2008), hyperventilation, restlessness, nausea or vomiting, loss of bowel or bladder control, rigidity or tremors, cyanosis, and convulsions (France et al. 2005). As Strong points out, participation in the ‘vital publics’ (2009: 173) of donation includes that ‘unique duty associated with the biological citizen’, the obligations and costs of the *munus*.

Waste is only one dimension of the moral register of cord blood donation. The registers of solidarism and universalism too come into conflict with a range of practical realities central to UCB banking. Donation is a highly charged form of moral identification and identity. Gillespie and Hillyer write of the ‘psychological commitment toward blood donation’ that can accumulate through the rhythms and cycles of regular donation (Gillespie and Hillyer 2002: 119). One poster on the wall of the Madrid blood and UCB bank discussed above reads: ‘*Salvas una vida y eres un heroe. Salvas tres y eres un donante*’ (‘Save a life and you're a hero. Save three and you're a donor’). Valentine (2005) explores a parallel discourse to that of waste in the ‘selflessness’ of the donor. ‘Sacrifice’ and ‘selflessness’ are particularly apt in a discussion about the void, the lack, the evisceration of self, at the heart of pure *communitas*.

And yet, the discourse of solidaristic universalism, together with the discourse of rights upon which it is premised, comes into conflict with the highly striated and exclusionary dimensions of the blood economies. As Valentine goes on to argue, for all the seemingly morally upright citizens who donate blood, there are those actively denied entry to the *communitas* of donors. Though there have been legal challenges, these exclusions can extend to gay and bisexual men, and their partners (until recently in the UK), the particularly old or young, sex workers, and those who have travelled to certain regions, etc. There are many categories of subject ineligible for citizenship within the *communitas* of donors. Blood donation ‘remains a public practice … that certain kinds of public are precluded from’ (*ibid*: 116). Indeed, political engagements around ‘individual rights vis-à-vis forms of institutional exclusion’ (Strong 2009: 172) highlight just how contradictory the *communitas* of blood can be.

For example, cord blood collection in the UK is for the most part confined geographically to those locations where there is a higher statistical probability of collecting from racial and ethnic minority populations (UK Stem Cell Strategic Forum 2010). Here, then, inclusion and exclusion are exercised by geographically concentrating the opportunity for donation in high density, racially heterogeneous cosmopolitan ‘world’ cities. And yet it is recognised how politically charged and volatile that process of selection and exclusion is:

*I don’t think it would have been ethical to say we’re not collecting from you. Because that might have been the only phenotype. Even in Caucasoids there are unique phenotypes. (Director of a public UCB bank 1)*

The realpolitik of British UCB donation unfolds in such a way that very few hospitals are actually resourced to undertake collection. The discourse of wastefulness, the void that must be filled, is the basis for an imperative obligation to donate that cannot itself be realised. In this way, the selectivity and discrimination of collection is in tension with expectations of universalism, of national solidarism and citizenship. While the charge or allegation of waste may be uniformly applied to the whole of the community, the opportunity to give, to replenish, to restore, is highly discriminating.

This exclusionary policing insists that donors practise a particular kind of living where race, sexual partners, nutrition, iron levels, geographic excursions are transposed onto participation in vital citizenship. The ‘costs’ of sustaining one’s relation to *communitas*, its selflessness, has an intrinsically ascetic self-denial at its core (Copeman 2009). The policing of eligibility problematises an understanding of blood donation as the ultimate inclusive ‘participatory space of belonging’ (Valentine 2005: 115) and challenges us to rethink public blood donation beyond an act both of, and for, community.

## Conclusion

The emerging picture of cord blood banking developed in this paper is one not easily categorised with reference to the bioethical and policy distinctions of gifts and commodities, the public and private, community and immunity. In the first place, the UCB banking world described here is one characterised by a highly distributed and globally scattered cosmopolitanism. Optimising the statistical probabilities of matching one immunity to that of a distant other cannot operate within the confined limits of the nation nor indeed locally embedded sentiments of solidarism. In other words, blood can be seen to become detached from *communitas* and projected into the dispersed immunitary worlds of internationalised migration and population movement. In Esposito’s terms, in becoming loosened from its *communitas*, cord blood enters an immunitary paradigm becoming ‘the power to preserve life’ (2008, 46).

UCB also calls into question traditional accounts of the ‘free’ circulation of gifts, of public domains insulated from markets. Units attract monetary value expressed through pricing, cost and reimbursement between trading partners domestically and internationally. This may possibly involve some notions of financialisation but without necessarily always involving a profit as such. To this extent, much of the UCB banking sector described here subverts both the public and the private, falling between the commons and the market.

Finally, we have critically reflected on the framing of cord blood as ‘waste’, as a form of relinquishment that implies little or no cost to the donor. We have shown that the process of extraction is far from being of little consequence to those considering UCB banking. Donors may find themselves caught between competing registers of the value and utility of umbilical cord blood both to the community and to their own neonate. Esposito might express this in terms of the problematic tension between that of *communitas* and *immunitas*, between the gift and the exemption. Further, the implied universalism underpinning solidaristic donation also fragments and splinters under highly variable conditions of opportunity and exclusion surrounding the practical organisation and legal governance of donation.

In following cord blood through the kinds of worlds and circuits described here, it becomes possible to understand better some of the more novel features of contemporary bio-objects. Cord blood can be seen to become a focus for the representation and calculation of life operating at a highly distributed international scale (Vermeulen et al. 2012). It can be seen to confound various codes of biopolitical and scientific definition (Holmberg and Ideland 2012) and disturb taken-for-granted boundaries between community and immunity (Esposito 2011). Even ‘banked’ bio-objects, it is possible to see, are far from static and are instead in a constant state of potential or actual movement. Such states of flux may be both *material* – ‘non-static’ – whilst also *definitional* in terms of being ‘non-axiomatic’ (Eriksson 2012).

The world of UCB discussed here, together with the wider blood economies, expresses highly variegated forms of hybrid practice that resist easy categorisation within much of the dominant bioethical and policy discourse on cord blood banking. However, it would be just as much a mistake to argue that all activities in the cord blood banking world are the same or that they operate on a flat terrain where distinctions and boundaries between activities, sectors, practices and logics have no meaning. Rather, we find hybrid zones of indistinction that may possibly require new reflection on the porosity or porousness of many traditional conceptions of the blood and tissue economies. Esposito’s argument is that an affirmative biopolitics is one rooted in just such an incompleteness and porosity of the borderlands of both the body and body politic. Bodies, both individual and collective, must resist and defend themselves, not against one another, but against closure and absolutist identification.

## Footnotes

1 This research was supported through a number of research projects funded by the UK Economic and Social Research Council: 2004–2007: ‘Haematopoietic Stem Cells: The Dynamics of Expectations in Innovation’ (Martin, Brown and Kraft - RES-340-25-0007); 2009–2010: ‘The political and moral economy of cord blood stem cell banking’ (Brown - RES-062-23-1386); 2012–2015: Post-graduate research funding for ‘Blood in the archive: rethinking the public umbilical cord blood ‘bank” (Williams - ES/J500215/1).
